# P071 Beyond the temporal arteries: a case of vertebral artery involvement detected by FDG-PET in giant cell arteritis

**DOI:** 10.1093/rap/rkag113.101

**Published:** 2026-11-03

**Authors:** Tony Yap, Mohammed Meher Ali Chaudhry, Kate Gralton, Thushyanthan Guruparan, Erica Gurung, Nadia Ahmad

**Affiliations:** Basingstoke and North Hampshire Hospital, Basingstoke, United Kingdom; Basingstoke and North Hampshire Hospital, Basingstoke, United Kingdom; Basingstoke and North Hampshire Hospital, Basingstoke, United Kingdom; Basingstoke and North Hampshire Hospital, Basingstoke, United Kingdom; Basingstoke and North Hampshire Hospital, Basingstoke, United Kingdom; Basingstoke and North Hampshire Hospital, Basingstoke, United Kingdom

## Abstract

**Introduction:**

Giant cell arteritis (GCA) has a heterogeneous clinical presentation depending on the vascular territories involved. Although headache, scalp tenderness, jaw claudication and visual symptoms are classical features, vertebral artery involvement is uncommon but clinically significant because of its association with posterior circulation ischaemia and stroke. We report a case of vertebral artery vasculitis identified by FDG-PET/CT in a patient presenting with typical cranial features of GCA despite negative temporal artery ultrasound and biopsy. Symptom recurrence with persistent dizziness during corticosteroid tapering prompted further investigation after MRI and MRA excluded intracranial pathology, leading to the diagnosis of large-vessel GCA.

**Case description:**

A 73-year-old female with a history of previous smoking, Fuchs endothelial corneal dystrophy, left central retinal vein occlusion (treated with intravitreal injections), and polymyalgia rheumatica (PMR) (on 1 mg prednisolone daily) presented with a three-week history of worsening temporal headaches along with left temporal, auricular and scalp tenderness on brushing her hair. There were no visual disturbances, jaw claudication or constitutional symptoms. She reported similar intermittent symptoms over the preceding three years. Examination demonstrated thickened temporal arteries with diminished pulses bilaterally. Of note, there were no carotid or subclavian bruits or focal neurological deficits at the time.

Inflammatory markers were mildly elevated at the time (CRP 7mg/L; ESR 26mm/hour) with normal levels three months prior. Connective tissue disease screen, rheumatoid factor and anti-cyclic citrullinated peptide antibodies were negative.

Given high clinical suspicion of GCA in the context of known PMR (GCA Probability Score 9 – intermediate risk), oral prednisolone 40 mg daily was commenced. Symptoms improved only partially. Temporal artery ultrasound and subsequent temporal artery biopsy were negative for GCA, prompting rapid tapering of glucocorticoids.

Persistent headache, dizziness and temporal artery tenderness, together with rising inflammatory markers following steroid withdrawal (CRP 20mg/L; ESR 23mm/hour), prompted further investigation. Cranial and cervical spine MRI and MR angiography (MRA) were then carried out. These excluded an acute stroke and did not demonstrate any features of vasculitis; however mild small-vessel disease, cervical spondylosis and sinusitis were identified. FDG-PET CT subsequently demonstrated increased bilateral vertebral artery uptake, consistent with vertebral artery vasculitis (Figure 1).

High-dose glucocorticoids were recommenced (prednisolone 40mg once daily for four weeks followed by a weaning regimen), resulting in complete resolution of symptoms and normalisation of inflammatory markers. Methotrexate was later introduced as a steroid-sparing agent.

**Discussion:**

Our case highlights several important learning points. Firstly, a high index of suspicion for GCA should be maintained in patients presenting with compatible cranial symptoms, elevated inflammatory markers and associated conditions such as polymyalgia rheumatica. However, vertebral artery involvement may present with non-specific neurological symptoms, making the diagnosis more challenging. Furthermore, the diagnostic performance of temporal artery ultrasound is operator-dependent, while the sensitivity of temporal artery biopsy may be reduced by skip lesions and prior glucocorticoid exposure. These limitations should prompt consideration of further vascular imaging when clinical suspicion remains high despite negative first-line investigations.

The 2023 EULAR imaging recommendations advocate ultrasound of the temporal and axillary arteries as the first-line investigation for suspected GCA, with FDG-PET/CT, MRI or CT angiography serving complementary roles in assessing extracranial large-vessel involvement. FDG-PET CT can be sensitive in cases where steroid doses are small and inflammatory markers remain elevated. In our patient, FDG-PET/CT proved pivotal in establishing the diagnosis of bilateral vertebral artery vasculitis after negative temporal artery ultrasound, biopsy and MRI/MRA. This supports current guidance recommending FDG-PET/CT when suspicion of large-vessel GCA persists despite inconclusive conventional investigations.

Finally, vertebral artery involvement, although uncommon, is clinically important because of its association with vertebrobasilar ischaemia and stroke. MRI and MRA remain essential to exclude intracranial pathology in patients presenting with posterior circulation symptoms, while FDG-PET/CT complements these modalities by detecting metabolically active vascular inflammation. This case highlights the importance of screening for extracranial large-vessel involvement to facilitate timely diagnosis and treatment.

**Key learning points:**

1. Giant cell arteritis should remain a key differential diagnosis in older patients presenting with compatible cranial symptoms, even when inflammatory markers are only modestly elevated or the presentation is atypical.

2. Negative temporal artery ultrasound and biopsy do not exclude GCA, particularly in patients with extracranial large-vessel involvement or prior glucocorticoid exposure. Persistent symptoms despite treatment should prompt reassessment of the diagnosis.

3. This case reinforces the 2023 EULAR imaging recommendations that advanced vascular imaging should be considered when first-line investigations are inconclusive but clinical suspicion of GCA remains high. FDG-PET/CT is particularly valuable for identifying extracranial large-vessel involvement, while MRI and MR angiography complement the diagnostic work-up by excluding posterior circulation stroke and other intracranial pathology.

4. Vertebral artery vasculitis is an uncommon but clinically important manifestation of GCA that may present with vertebrobasilar symptoms such as persistent dizziness.

5. MRI and MR angiography remain essential to exclude posterior circulation stroke and other intracranial pathology in patients presenting with neurological symptoms, complementing FDG-PET/CT in the diagnostic work-up.

6. Early recognition of vertebral artery involvement is crucial because delayed diagnosis may increase the risk of vertebrobasilar ischaemia and stroke, whereas prompt immunosuppressive treatment can prevent irreversible complications.

**P071 Figure 1:**
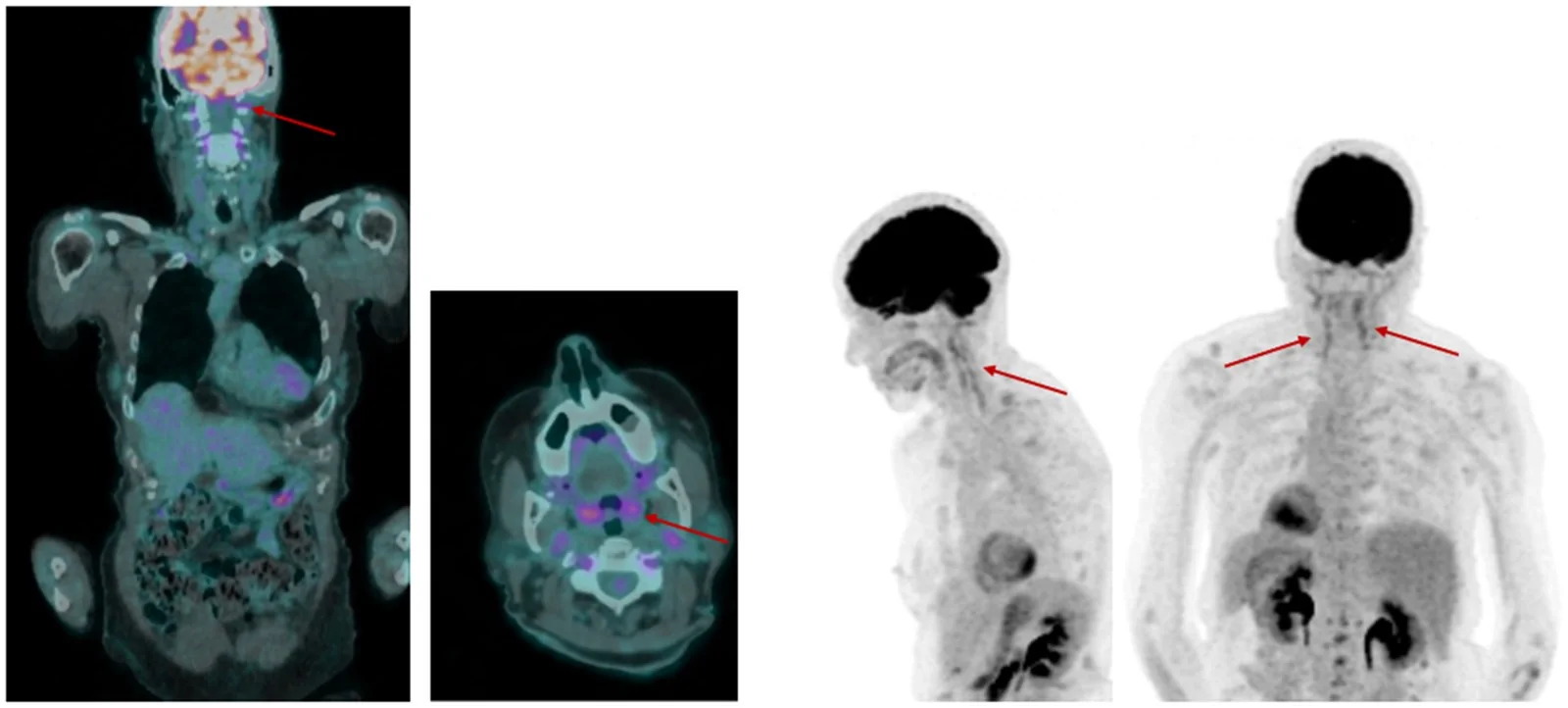
PET-CT showing increased FDG uptake in vertebral arteries bilaterally (red arrows)

